# Inter-facility travel time and referral for emergency obstetric care in the 15 most populated cities of Nigeria: a spatial analysis

**DOI:** 10.1186/s44263-026-00285-8

**Published:** 2026-05-28

**Authors:** Kerry L.M. Wong, Aduragbemi Banke-Thomas, Tope Olubodun, Uchenna Gwacham-Anisiobi, Abimbola Olaniran, Abiodun Amosu, Michael Ezeanochie, Bala Harri, Babagana Bako, Charlotte Stanton, Shravya Shetty, Gautam Prasad, Taiwo Amole, Daniel Achugo, Aminu Umar, Rosemary Ogu, Bosede B. Afolabi

**Affiliations:** 1 Faculty of Epidemiology and Population Health, London School of Hygiene and Tropical Medicine, London, UK; 2 School of Human Sciences, University of Greenwich, London, UK; 3 Maternal and Reproductive Health Research Collective, Lagos, Nigeria; 4 Department of Community Medicine and Primary Care, Federal Medical Centre Abeokuta, Abeokuta, Ogun Nigeria; 5 Nuffield Department of Population Health, University of Oxford, Oxford, UK; 6 KIT Royal Tropical Institute, Amsterdam, Netherlands; 7 Ministry of Defence Health Implementation Programme, Abuja, Nigeria; 8 Department of Obstetrics & Gynaecology, University of Benin Teaching Hospital, Benin City, Edo State Nigeria; 9 Department of Health Planning, Research and Statistics, Federal Ministry of Health, Abuja, Nigeria; 10 Department of Obstetrics and Gynaecology, University of Maiduguri, Maiduguri, Nigeria; 11 Google LLC, 1600 Amphitheatre Parkway, Mountain View, Mountain View, CA United States of America; 12 Department of Community Medicine, Bayero University Kano, Kano, Nigeria; 13 African Centre of Excellence for Population Health Policy, Bayero University Kano, Kano, Nigeria; 14 College of Health Sciences, Nnamdi Azikiwe University, Awka, Anambra State Nigeria; 15 School of Medicine, Keele University, Keele, Staffordshire, UK; 16 Department of Obstetrics and Gynaecology, University of Port Harcourt, Rivers, Nigeria; 17 Department of Obstetrics and Gynaecology, College of Medicine, University of Lagos, Lagos, Nigeria

**Keywords:** Inter-facility travel time, emergency obstetric care, health service, referral, Nigeria, Geospatial

## Abstract

**Background:**

Timely access to comprehensive emergency obstetric care (CEmOC) can be vital for ensuring maternal and newborn survival. However, pregnant women in need of CEmOC may not initially present at a CEmOC facility, thereby necessitating inter-facility referral. We assessed inter-facility travel time between potential referring non-CEmOC facilities and receiving CEmOC facilities in the 15 largest Nigerian cities.

**Methods:**

Data was sourced from the 2018 Nigeria Health Facility Registry, with additional facilities verified in 2022. We applied Google Maps Platform’s internal Directions Application Programming Interface (API) to derive driving times from each 600m^2^ S2 cell to their respective nearest CEmOC facilities. Geographic coordinates of non-CEmOC facilities were mapped to S2 cells to retrieve travel time to public CEmOC facilities. Travel times were estimated from each S2 cell to the nearest CEmOC facilities by ownership (public and private) under peak traffic scenario (weekdays 18–20 h) and off-peak traffic scenario (weekends 01–03 h). Based on the shortest inter-facility travel time, each non-CEmOC facility was paired with a public CEmOC facility. Median travel time and percentage of non-CEmOC facilities located > 30 and > 60 min to the nearest public CEmOC facility were estimated. Sensitivity analysis comparing the API’s travel time estimates for randomly-selected 10% of non-CEmOC facilities with those from other methods was conducted.

**Results:**

Altogether, 4,563 and 1,963 non-CEmOC and CEmOC facilities were included, respectively. Percentage of non-CEmOC facilities located > 30 min to the nearest public CEmOC was highest in Port Harcourt (51%) and lowest in Maiduguri (6%). All non-CEmOC facilities were located ≤ 60 min from the nearest public CEmOC facility in Aba, Owerri, and Ilorin. Median number of non-CEmOC facilities connected to a public CEmOC facility was 27, with five public CEmOC facilities connected to > 100 non-CEmOC facilities. For some non-CEmOC facilities, the nearest public CEmOC facilities are in a contiguous city or state.

**Conclusions:**

Inter-facility referrals in large Nigerian cities show substantial variation in travel time and uneven referral loads, revealing critical pressure points that may delay timely access to CEmOC. Integrating travel time metrics into maternal health planning is essential for improving the efficiency, equity, and resilience of resource-constrained urban referral systems.

**Supplementary Information:**

The online version contains supplementary material available at 10.1186/s44263-026-00285-8.

## Background

About 260,000 women lost their lives during pregnancy and childbirth in 2023, almost 70% occurred in sub-Saharan Africa (SSA). Similarly, 44% of the estimated two million stillbirths in 2023 took place in SSA. Worldwide, Nigeria has the highest number of maternal deaths (75,000 [29%]) and the third highest number of stillbirths (183,749 [9%]) [1, 2]. Half of maternal deaths and three-quarters of intrapartum stillbirths are preventable with timely access to high-quality emergency obstetric care (EmOC) at functioning and capable facilities [3].

EmOC is a package of evidence-based clinical and surgical interventions (also known as signal functions) provided by skilled healthcare personnel. Facilities that provide EmOC can be basic emergency obstetric care (i.e. BEmOC meaning such facilities provide injectable antibiotics, injectable oxytocics, anticonvulsants, manual removal of placenta, removal of retained products, assisted vaginal delivery and neonatal resuscitation) or comprehensive emergency obstetric care (i.e. CEmOC which can provide all BEmOC interventions plus blood transfusion and caesarean section). In most health systems, EmOC provision is delivered across three tiers: tertiary (e.g., national referral hospitals), secondary (e.g., general hospitals), and primary (e.g., health centres). The tertiary and secondary facilities are typically CEmOC facilities located in urban areas while primary facilities tend to operate at below EmOC level in both rural and urban areas [4].

With an annual 1.8% urban population increase since 1990, two-thirds of the world’s population will live in urban settings by 2050, with a projected additional 1.25 billion people concentrated in African cities [5]. This increased urban population will put pressure on already overstretched resources in urban SSA. Adding to this pressure are the pregnant women from rural areas needing CEmOC who would have to travel to urban areas for appropriate care [6]. Such rapid population and health need growth strain limited resources and makes management and health planning highly challenging in cities and surrounding suburbs. Yet even in cities, not all secondary and tertiary facilities have the capacity and mandate to provide the full suite of CEmOC interventions [7]. This means that pregnant women might need inter-facility referrals [8], causing delays in reaching definitive care [9]. As obstetric complications are often unpredictable and offer a short time window to start the required level of care, it is paramount that inter-facility referrals are effective and optimised such that women presenting at the first facility can be efficiently referred to CEmOC facilities when complications may necessitate this. As in other similar low-resource urban settings, journeys of pregnant women living in highly populated Nigerian cities, are typically characterised by heavy traffic and poor road conditions. Slum dwellers experience disproportionate inequities in reaching EmOC facilities compared to other urban residents [10, 11].

In 2020, the United Nations Population Fund published guidelines for optimising referral networks for EmOC service provision targeted at increasing geographical coverage and improving quality of care [12]. Two critical considerations to ensure effective referral are travel time and the means of transport to reach a CEmOC facility. Existing evidence shows that pregnant women with inter-facility travel time of 30 min have significantly higher odds of maternal death compared to those who travelled less [13]. Furthermore, babies who are referred, irrespective of the inter-facility travel time, face increased odds of stillbirth compared to those not referred [14].

Accurate measures of inter-facility travel time are critical for strengthening EmOC referral systems. To date, most studies accessing inter-facility travel time are based on modelling approaches like cost-friction approach with model performances dependent on high-quality observational data and parameterization [15–17]. Such dependences make model outputs insufficient in capturing the lived experience of travel to care, more so in urban settings where traffic congestion, sprawling urban slums, urban swell, and a plethora of dysfunctional private providers exist [18, 19]. Fluctuating populations moving from surrounding residential areas around the city to the city proper during the daytime and returning afterwards have also been reported. This means variation in geographical accessibility to critical services such as EmOC in cities during peak and non-peak times [11]. Navigation applications such as Google Maps (Google LLC, Mountain View, California, US) collect real-time data from road users and can account for peak and non-peak travel time variations [11]. In Lagos, Nigeria, Google Maps travel times estimates have been found to approximate observed travel times to functional health facilities more closely than some alternative approaches, such as cost-friction models [18]. In this study, we aimed to assess inter-facility travel time from all potential referring facilities without the capacity to provide CEmOC (referred to as non-CEmOC facilities) to receiving facilities capable of providing such care (CEmOC facilities) using travel time estimates from Google Maps. We also assessed the connectivity between the non-CEmOC and CEmOC facilities based on a referral network that places emphasis on minimising travel time rather than administrative boundaries.

## Methods

### Study design and setting

This cross-sectional spatial analysis was set in Nigeria, and involved city boundary delineating, verification of health facilities’ geographical location, functionality and capacity to provide CEmOC and travel time estimates derived from the Google Maps Platform’s internal Directions API (Google LLC, Mountain View, California, US). Nigeria is administratively divided into 36 states and a Federal Capital Territory. The states are divided into 774 local government areas (LGAs). Each state has one or two major cities, with at least 20 of the major cities across the country. We conducted this study in 15 Nigerian cities (Aba, Abuja, Benin City, Ibadan, Jos, Kaduna, Kano, Lagos, Ilorin, Maiduguri, Onitsha, Owerri, Port Harcourt, Uyo, and Warri). Each has an estimated population in 2022 or projected population by 2030 (end term of the Sustainable Development Goals (SDGs)) of at least one million. Overall, the 15 cities accounted for 26% of the national population in 2022. Across these cities, most tertiary facilities are owned and funded by the Federal government while secondary facilities are owned and funded by the state government. Available evidence from Nigeria shows that nine in 10 pregnant women travel to the receiving facility by motorised transport, with less than 10% using an ambulance [20, 21].

### Data sources

As the selected cities lacked clearly defined boundary lines that demarcated their urban extent, needed for the planned quantitative and spatial analysis, we established the boundary of each city (including suburbs) by the LGAs that make it up. The vector file of the LGA boundaries [22], WorldPop’s gridded surface of the population (at 100m^2^ resolution) [23], Google Maps and Global Human Settlement (GHS) layers showing the gridded surfaces of urban areas [24] were spatially superimposed. LGAs containing areas of higher population density than their surroundings or marked as urban or suburban/peri-urban in the GHS layer were selected [25]. Inputs from co-authors who were familiar with the cities facilitated the demarcation process. We then considered the demarcated areas as comprising of level 14 S2 cells (approximately 600m^2^ in size), a spatial indexing method used by Google to divide spherical surfaces into grids of approximately equal size [26]. This resolution was selected to ensure that there was a balance between precision and computational power.

The official national geocoded health facility master list is available in the 2018 Nigeria Health Facility Registry (NHFR) [27]. We extracted data on all hospitals in the 15 included cities from the NHFR, including facility name, ownership (public (federal or state) or private (for-profit, not-for-profit, faith-based organisations, military, or police-owned)), location (LGA and GPS coordinates), and operational status (open or closed). The NHFR data was supplemented by state-specific lists (e.g., Health Facilities Monitoring and Accreditation Agency in Lagos State), snowballing technique, and data gathered from stakeholders familiar with health service provision at the state level. We consolidated the various lists, de-duplicated, and assigned unique identifiers to each hospital. In doing this, we identified and harmonised facilities with multiple names where these were different on the NHFR and on other state-specific lists. Data on service availability was obtained through a facility functionality assessment survey conducted with health facility administrators to establish CEmOC functionality. The survey was conducted during in-person hospital visitations by trained research assistants using a short questionnaire which we developed for this study [28]. The questionnaire was administered to all health facility managers who voluntarily decided to participate in the study. Data collection took place from March to August 2022, and the final dataset is publicly available on Figshare [29] (Figshare LLP, London, United Kingdom).

From the survey, facilities were classed as CEmOC facilities, if they were operational 24 h a day and able to conduct caesarean sections (used as a proxy for CEmOC in this study, as capacity for other EmOC services provision is usually subsumed in capacity for caesarean Sects. [4, 30]). On the other hand, for this analysis, non-CEmOC facilities were defined as hospitals assessed as not able to perform caesarean sections at all or not able to perform it 24/7. In addition, we included all facilities classified as primary level facilities in NHFR as non-CEmOC facilities as they are not expected to provide caesarean sections. The classification of facilities into CEmOC and non-CEmOC facilities was reviewed by local experts and the full list of verified CEmOC facilities included in this study has been made publicly available [28].

Health facilities in the NHFR with valid geographic coordinates recorded as latitude and longitude were spatially joint with our city demarcation. In the current analysis, non-CEmOC facilities with coordinates located inside the demarcated area, and CEmOC facilities that were located inside or near the demarcated area were included.

### Inter-facility travel time for emergency obstetric care referral

Next, we conducted backend extraction of travel times to CEmOC facilities from the API for the level 14 S2 grid cells (Supplementary Material 1: Fig S1). The API uses machine learning to estimate travel time (Supplementary Material 1 details methods used by the API to estimate travel time). For this analysis, we estimated travel time based on motorised private transport, being the commonest mode of transport for pregnant women in emergency in Nigeria [20, 21]. Travel times estimates from each grid centre to the nearest CEmOC facilities by ownership (public and private) under peak traffic scenario (weekdays 18–20 h) and off-peak traffic scenario (weekends 01–03 h) were obtained using the Google Maps API. The selection of 18–20 h period to represent peak traffic conditions was based on data generated from the Google Maps API and available evidence on period of the day that falls within the broader evening congestion window commonly experienced across major Nigerian cities [11, 28, 31]. Latitude and longitude coordinates of the non-CEmOC facilities were joined to their nearest or encapsulating S2 grid cell in order to obtain metrics on inter-facility travel going to CEmOC facilities. A full dataset enlisting all S2-cell level estimates has been published [28, 32]. We also obtained the number of public CEmOC facilities reachable within 15, 30, and 60 min and the facility identifiers of up to three nearest public CEmOC facilities.

The assessment of the pattern of network and connectivity underlying non-CEmOC facilities and public CEmOC facilities was operationalised by pairing each non-CEmOC facility with a public CEmOC facility based on the shortest travel time required. A similar approach to identify pairs of referring facility (non-CEmOC facilities) and receiving facilities (CEmOC facilities) was used in a previous study conducted in Tanzania [17]. The current analysis was limited to public CEmOC facilities, where most births occur [33], thereby providing insights most relevant to public health decision making.

### Data analysis

For each city, we summarised the number of non-CEmOC facilities by the travel time thresholds to the nearest public CEmOC facilities. Further, median travel time (MTT) to the nearest, second nearest, and third nearest public CEmOC facilities from public non-CEmOC facilities, private non-CEmOC facilities, and all non-CEmOC facilities were obtained. By city, the median number of reachable public CEmOC facilities under the three different time thresholds (15, 30, and 60 min) for peak time traffic from any public non-CEmOC facility, any private non-CEmOC facility, and any non-CEmOC facility were also presented. For each public CEmOC facility, the number of paired/connected non-CEmOC facilities were plotted. Lastly, maps showing all non-CEmOC facilities and their respective nearest (requiring the shortest travel time to reach) public CEmOC facilities were drawn. We used these maps to visualize the configuration underlying existing emergency obstetric referrals. Researchers familiar with the local context reviewed these illustrative maps. Analysis and visualisation as static maps in this study were done with R version 4.3.2 (R Core Team, Vienna, Austria).

To provide the ground to assessing the sensitivity of our findings to the choice of travel-time model, we compared Google Maps API travel time estimates to the nearest public CEmOC facility for randomly-selected 10% of the non-CEmOC facilities with those obtained from Open Source Routing Machine (OSRM) and a cost-friction surface approach, using the *OSRM* and *traveltime* packages in R [34]. OSRM travel time were set to motorised from of travel, and the shortest time from the OSRM approach was obtained from all possible pair-wise combinations within each city. The global friction surfaces for motorised travel for the year 2020 generated by Weiss and colleagues was used [35].

All the codes used in this analysis are publicly available in Figshare [36].

## Results

### Summary characteristics of included non-CEmOC and CEmOC facilities

This study was conducted in 15 cities, spanning 106 LGAs of Nigeria. The number of non-CEmOC facilities included was 4,563, with 2,587 (57%) in the public sector. Among public non-CEmOC facilities, 103 (4%) were classified as secondary or tertiary facilities from NHFR where CEmOC provision was not available at the time of the assessment. In Maiduguri, 11% (9 of 84) of public secondary/tertiary facilities did not provide CEmOC. Among 1,976 private non-CEmOC facilities, 30% were classified as secondary or tertiary but did not provide CEmOC. In Uyo, 46 of 48 private non-CEmOC facilities were classified as secondary/tertiary (Table 1). In all instances, public tertiary facilities assessed to be non-CEmOC were assessed as such because they are specialist hospitals not designed to provide obstetric care. This included Federal Neuro-Psychiatric Hospital (Maiduguri), Barau Dikko Paediatric Hospital (also known as Barau Dikko Teaching Hospital) (Kaduna), and National Orthopaedic Hospital (Lagos). For secondary facilities, reasons for non-CEmOC classification included specialist hospitals not designed to provide obstetric care, the non-availability of personnel to provide caesarean section, the lack of functioning theatre and the non-availability of blood and blood products.

**Table 1:** The distribution of non-CEmOC and CEmOC facilities across 15 cities in Nigeria

State	City	Non-CEmOC facilities							CEmOC facilities			Women of Childbearing Age in 2022
		Public non-CEmOC facilities			Private non-CEmOC facilities			All non-CEmOC facilities				
		Total count	Classified as Secondary (Tertiary) in the National Health Facility Register	Percentage classified as Secondary or Tertiary in the National Health Facility Register	Total count	Classified as Secondary in the National Health Facility Register	Percentage classified as Secondary in the National Health Facility Register		Public CEmOC facilities	Private CEmOC facilities	All CEmOC facilities	
Imo	Owerri	27	1	4%	37	22	59%	64	2	61	63	230314
Borno	Maiduguri	84	8 (1)	11%	24	17	71%	108	5	21	26	265740
Plateau	Jos	204	1	0%	60	5	8%	264	6	70	76	344494
Abia	Aba	145	1	1%	55	23	42%	200	2	93	95	377554
Anambra	Onitsha	110	3	3%	111	80	72%	221	2	102	104	397541
Akwa Ibom	Uyo	147	8	5%	48	46	96%	195	3	44	47	461254
Edo	Benin City	133	4 (1)	4%	160	32	20%	293	2	68	70	482055
Kaduna	Kaduna	160	4 (1)	3%	104	40	38%	264	5	46	51	513105
Delta	Warri	156	13	8%	55	6	11%	211	9	100	109	567385
Kwara	Ilorin	82	4 (1)	6%	43	22	51%	125	7	63	70	583066
Rivers	Port Harcourt	128	8	6%	17	6	35%	145	5	79	84	828146
Oyo	Ibadan	280	17 (1)	6%	153	112	73%	433	10	153	163	955580
FCT	Abuja	244	3	1%	186	41	22%	430	18	51	69	1095195
Kano	Kano	400	13 (2)	4%	97	29	30%	497	16	129	145	1294941
Lagos	Lagos	287	7 (1)	3%	826	104	13%	1113	26	765	792	3402451
Total		2587	103 (8)	4%	1976	585	30%	**4563**	118	1845	**1963**	11798821

**Fig. 1:**
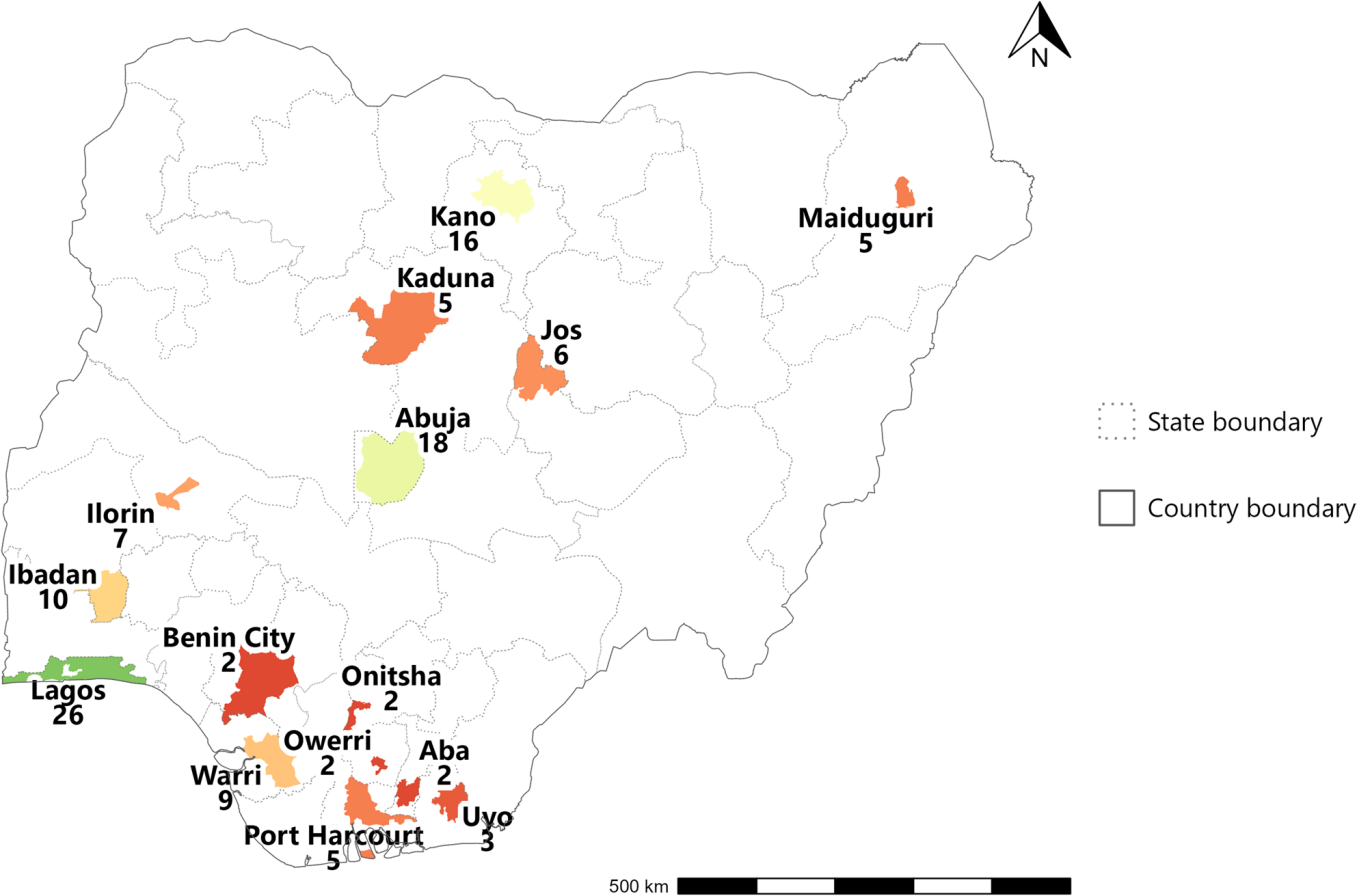
Distribution of 118 public CEmOC facilities across 15 cities in Nigeria

Across all cities, 1963 CEmOC facilities were verified, with 118 (6%) being in the public sector (Table 1; Fig. 1). The largest number of public CEmOC facilities were seen in Lagos, followed by Abuja and Kano (26, 18, and 16, respectively). In four cities (Aba, Benin City, Onitsha and Owerri), the number of public CEmOC facilities was below 3 (Table 1).

### Travel time from non-CEmOC facilities to the nearest public CEmOC facility

In Port Harcourt, travel time to the nearest public CEmOC exceeded 30 min in 51% non-CEmOC facilities, majority (96%) of the non-CEmOC facilities exceeding the 30-minute travel time mark were public sector facilities. In Aba, Jos, Uyo, Abuja, and Benin City, more than one-third of non-CEmOC facilities exceeded the 30-minute threshold. In Kano and Maiduguri, all non-CEmOC facilities more than 30 min from public CEmOC were in the public sector. On the other hand, in Owerri and Lagos more than half of the facilities over 30 min from public CEmOC were private facilities. By count, over 100 non-CEmOC facilities were located over 30 min to their nearest public CEmOC facilities in Jos, Abuja, Benin City, Kano, and Lagos. Travel time to the nearest public CEmOC exceeded 60 min in more than 10% of non-CEmOC facilities in Port Harcourt (17%), Jos (15%), and Benin City (12%). In Jos, 26% of non-CEmOC facilities located over 60 min to the nearest public CEmOC facilities were privately owned (Table 2).

**Table 2:** Percentage of any non-CEmOC facilities located over 30 and 60 min to the nearest public CEmOC facility

City	Number of non-CEmOC facilities	Percentage of non-CEmOC facilities located > 30 min to the nearest public CEmOC (%)	Percentage of public sector facilities out of those located > 30 min to the nearest public CEmOC (%)	Percentage of non-CEmOC facilities located > 60 min to the nearest public CEmOC (%)	Percentage of public sector facilities out of those located > 60 min to the nearest public CEmOC (%)
Port Harcourt	145	51	96	17	100
Aba	200	46	78	0	-
Jos	264	41	83	15	74
Uyo	195	36	89	2	100
Abuja	430	35	73	3	100
Benin City	293	34	88	12	89
Kaduna	264	28	89	9	96
Owerri	64	22	43	0	-
Kano	497	20	100	1	100
Ibadan	433	19	83	1	100
Lagos	1113	15	44	3	59
Onitsha	221	14	87	4	100
Warri	211	12	85	4	100
Ilorin	125	12	80	0	-
Maiduguri	108	6	100	4	100
**Total**	**4563**	**24**	**79**	**4**	**87**

Footnote: CEmOC: Comprehensive Emergency Obstetric Care

**Fig. 2:**
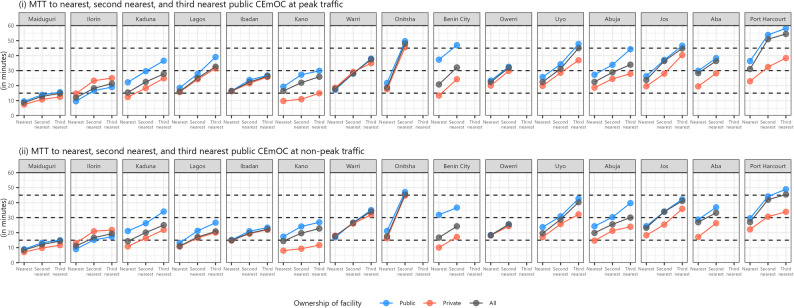
Median travel time (MTT) from any non-CEmOC facilities to the nearest, second nearest, and third nearest public CEmOC facilities

At both peak and non-peak traffic, MTT to the three nearest public CEmOC facilities from all (public and private) non-CEmOC facilities were within 45 min in all cities but Port Harcourt (58 min). From public non-CEmOC facilities, travel time to the nearest public CEmOC facility was below 30 min in 13 of 15 cities (36 min in Port Harcourt and 37 min in Benin City) on average (Fig. 2).

Compared to in peak traffic conditions, travel time in non-peak traffic conditions were largely similar in most cities, other than Lagos and Jos. MTT from private non-CEmOC facilities to public CEmOC facilities were considerably shorter in Kaduna, Kano, Benin City, Abuja, and Port Harcourt; and the two were very similar in Ibadan and Warri. In Ilorin, MTT to public CEmOC facilities were shorter from public non-CEmOC than from private non-CEmOC (Fig. 2).

### Number of public CEmOC facilities reachable within 15, 30, and 60 min

**Fig. 3:**
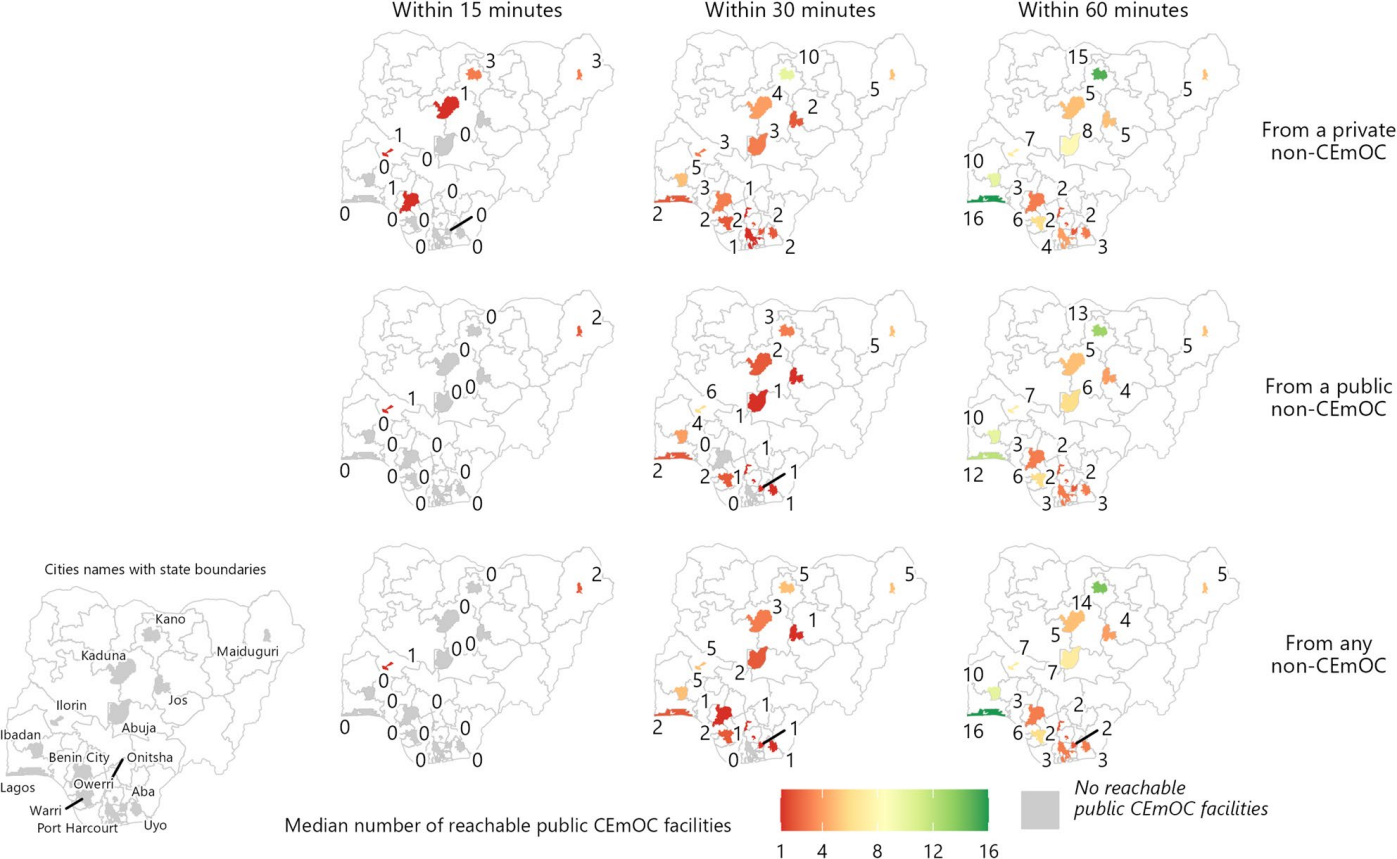
Median number of public CEmOC reachable under different time thresholds

Using the 15-minute time threshold, the median number of public CEmOC reachable from public non-CEmOC was one in Ilorin, two in Maiduguri, and zero elsewhere. Using the 30-minute time threshold, the number of public CEmOC reachable from public non-CEmOC was zero in two cities (Benin City and Port Harcourt). Private non-CEmOC facilities were better connected to public CEmOC in all cities. On the other hand, the number of public CEmOC reachable within 30 min from public non-CEmOC were the highest in Ilorin (6), Maiduguri (5), and Ibadan (4); the number of public CEmOC reachable within 30 min from private non-CEmOC were highest in Kano (10), followed by Ibadan (5) and Maiduguri (5). As the time threshold increased to 60 min, the number of public CEmOC reachable increased most substantially in Lagos (from two to 16) and Kano (from five to 14) (Fig. 3).

### Non-CEmOC and public CEmOC facilities by the shortest travel time

**Fig. 4:**
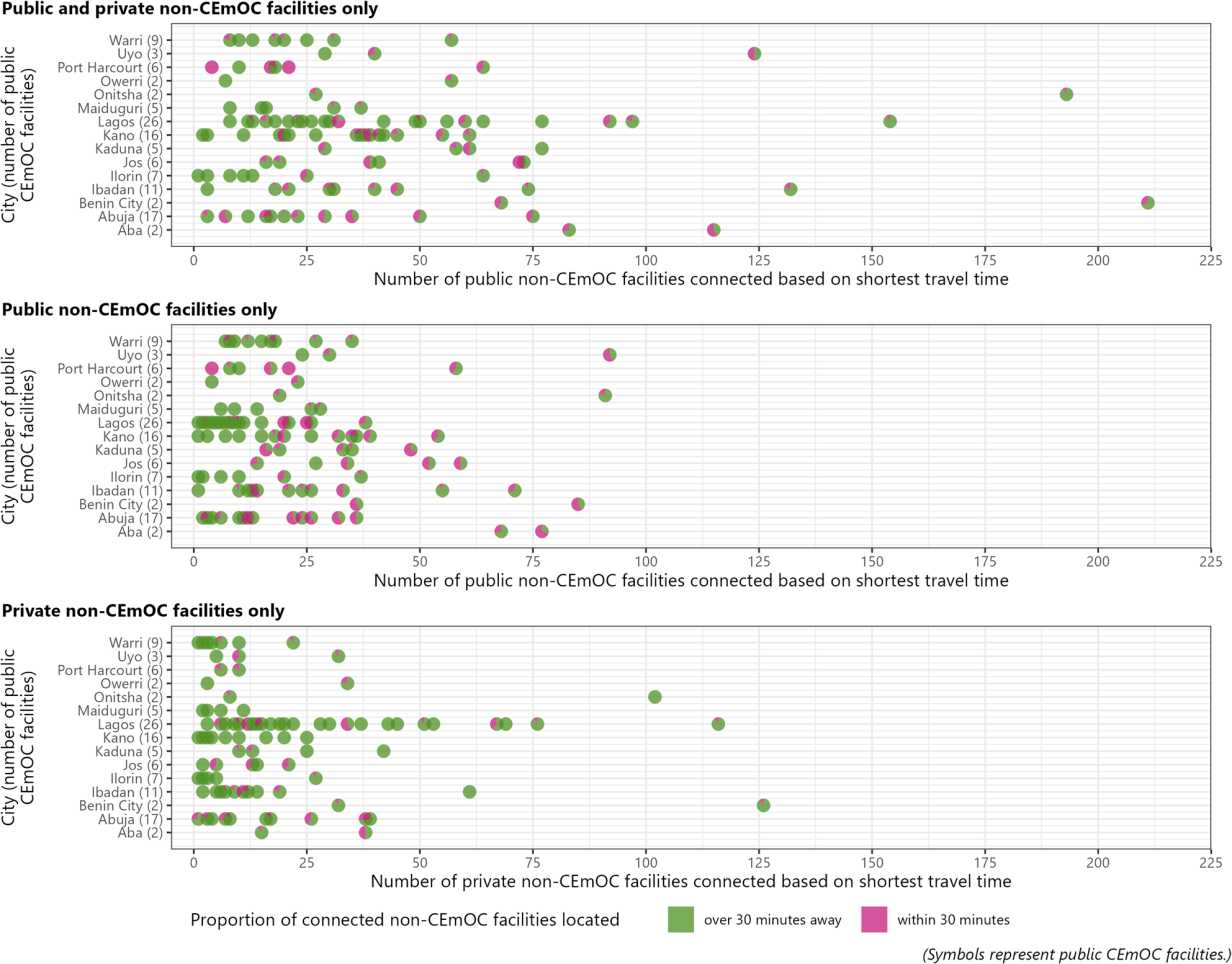
The number of connected non-CEmOC facilities per public CEmOC facility, based on the shortest travel time from non-CEmOC facility to public CEmOC facility

Each non-CEmOC facility was linked to their respective nearest public CEmOC facility, as guided by minimising inter-facility travel time (Fig. 4). Across 119 public CEmOC facilities, the median number of connected facilities was 27. In five public CEmOC facilities, the number of connected non-CEmOC facilities exceeded 100. All of these were in the south, with Central Hospital Benin City in Benin City identified as being the nearest to the highest number of non-CEmOC facilities (212), 30% of which were located over 30 min away. In 18 public CEmOC facilities, at least 50% of the connected non-CEmOC facilities were located over 30 min away.

In Onitsha, Nnamdi Azikiwe University Teaching Hospital is the nearest to 27 non-CEmOC facilities – eight public and 19 private – by travel time, whilst Onitsha General Hospital is the nearest to 193 non-CEmOC facilities,102 and 91 public and private non-CEmOC facilities, respectively. The spatial network of inter-facility connectivity based on shortest travel time were presented in Supplementary Material 2: Fig. S1-10. In Port Harcourt, Onitsha, Benin City, and Warri, the nearest public CEmOC facility to some non-CEmOC facilities were in a contiguous city or even state (Supplementary Material 2: Fig S1 and Fig S2).

In Abuja, the 16 public CEmOC facilities were relatively spread out. In the southwest, Kwali General Hospital was the nearest public CEmOC facility to 35 non-CEmOC facilities, some of which were also within 30 min to Asokoro General Hospital (nearest public CEmOC facilities for 3 non-CEmOC facilities) (Supplementary Material 2: Fig S4). This pattern was also observed elsewhere, including, for example, in Kano (Supplementary Material 2: Fig S8).

For a random sample of 10% of the non-CEmOC facilities, estimates of travel time to the nearest public CEmOC facility obtained from OSRM and cost-friction surface approach are presented in Supplementary Material 3. The overall median time from these sampled facilities to the nearest public CEmOC facility from Google Maps API was 19 min, as opposed to 7 and 5 min from OSRM and cost-friction surface approach, respectively.

## Discussion

We performed a comprehensive assessment of the geo-accessibility between all non-CEmOC facilities and their nearest public CEmOC facilities in the 15 most-populated cities in Nigeria. Across all cities, the percentage of non-CEmOC facilities needing > 30 min to reach the nearest public CEmOC was highest in Port Harcourt (51%) and lowest in Maiduguri (6%). The majority of the non-CEmOC facilities located > 30 min to the nearest public CEmOC were public sector facilities (except in Owerri). Going from public non-CEmOC facilities, average travel time to reach the nearest public CEmOC facility was below 30 min in 13 of 15 cities. In addition, our results show that peak and non-peak traffic conditions did not pose substantial difference in inter-facility travel time.

Considering inter-facility travel time, we did not see the north-south divide often seen in other domains of inequalities in Nigeria, including in healthcare and health outcome [37, 38]. This suggests that inter-facility travel time probably has more to do with the unique infrastructural distribution of cities. In contrast, we observed a north-south difference in the number of public CEmOC facilities reachable from non-CEmOC facilities within shorter travel time thresholds. Specifically, there were more reachable public CEmOC facilities within shorter travel times in northern cities (e.g., Kaduna, Kano, and Maiduguri) compared to southern cities (e.g., Aba, Port Harcourt, Owerri, and Uyo). This pattern may reflect the clustering of CEmOC facilities and road networks in certain northern urban centres, despite the generally denser distribution of infrastructure in southern cities. The most populated city, Lagos, had substantial number of public CEmOC facilities reachable only at 60 min of travel while the second most populated, Kano, had 5 within 30 min from any non-CEmOC).

Assessing the number of ‘connected’ non-CEmOC facilities to the 119 public CEmOC facilities based the shortest travel time, we found a varied picture. The most extreme scenario was identified in Onitsha, where the two public CEmOC facilities were connected to 27 and 194 non-CEmOC facilities, respectively. Across all cities, five public CEmOC facilities had more than 100 connected non-CEmOC facilities. This raises concerns about uneven distribution of potential patient volume that such facilities will be receiving. For some public CEmOC facilities, all connecting non-CEmOC facilities were located over 30 min away. Also, many receiving facilities (public CEmOC) appear to be the first points to many referring (non-CEmOC) facilities that are also within reasonable proximity to other receiving facilities, indicating the availability of an option range. Evidencing the tens and hundreds of public and private non-CEmOC facilities feeding into some public CEmOC facilities points urgently to the need to unpack the intricacies of CEmOC referrals against the backdrop of these complicated cities.

To our knowledge, this is the first large-scale effort mapping out health facilities by their capacity to provide CEmOC and comprehensively assessing referrals from all non-CEmOC facilities to receiving facilities in urban settings. Albeit unlikely the full mechanism driving facilities connectivity and inter-facility referrals, inspecting the shortest travel time between pairs of non-CEmOC and CEmOC facilities offers insight into the facility network, if travel time efficiency and minimal delay were considered the top criteria [12]. In addition, we used travel time estimates from a navigation software, Google Maps, which has been shown to generate more realistic estimates of travel time compared to modelled approaches such as cost-friction in a previous study conducted in Lagos, the largest megacity in Africa [18].

However, several limitations should be considered. First, we did not account for bed capacity at CEmOC facilities. Future data collection efforts should consider indicator of resource capacity to enable better understanding of care provision. Second, our exclusion of private CEmOC facilities, often preferrable to some pregnant women due to perceived better treatment [39, 40], as receiving facilities, may have led to an over-estimation of travel time. However, this exclusion was motivated by the need to eliminate the likely financial hinderance to access appropriate care that is usually posed by private sector facilities. Previous studies have also excluded private facilities [41, 42]. Third, our health facility data collection was concluded in 2022. Recent infrastructural changes, including the constructions of new roads, bridges, flyovers, health facilities, may have altered inter-facility travels and consequently travel time. In Benin City, for instance, two more public CEmOC facilities have been renovated (Central Hospital Benin city and Stella Obasanjo Hospital for women and Children) and one private CEmOC facility (Merry Ehanire Hospital for Women and Children) have become operational since our data collection process [43, 44]. Further, facilities not registered on the NHFR are not captured, likely affecting private sector facilities to a greater extent. Fourth, our identification of non-CEmOC facilities was partly based on the level of care classification in the NHFR [27], though the database is not immune to misclassification [45], which could have impacted our estimates. However, the risk of misclassification bias was potentially limited by the extensive review of the entire panel of facilities for each state by experts local to the state. Fifth, we have used travel time data from Google Maps, which, although openly accessible [28], is generated by a proprietary platform with limited algorithmic transparency compared with alternative methods such as cost friction and OSRM. Despite this limitation, Google Maps is widely used in health service research [11, 46–49]. In our comparison based on 10% random subsample, non-proprietary methods produced substantially shorter inter-facility travel times than Google Maps, indicating a systematic discrepancy. This is consistent with previous work showing that Google Maps yields longer and more traffic-aware travel times that are closer to observed journeys than cost-friction and OSRM, which approximate near–free-flow conditions using assumed speeds [50]. In dense urban settings, where Google Maps’ incorporation of observed traffic conditions and congestion likely produce more realistic and policy-ready estimates of driving time. On the other hand, cost friction and OSRM may be more accurate in areas with limited mobile phone coverage or where road networks and traffic conditions are less well represented [51]. Policy-relevant quantities such as the proportion of facilities exceeding 30- or 60-minute thresholds therefore depend on the chosen travel-time model, and modelled estimates should be calibrated and, where necessary, debiased using context-specific empirical travel data, as underestimation of true travel time for CEmOC referral can have significant implications for maternal and perinatal survival. Sixth, the Google Directions API uses crowdsourced GPS data and live traffic conditions to produce a single point estimate without uncertainty bounds. The travel time estimates reported in this study are based on both peak and off-peak conditions, partially capturing variability by traffic volume and thereby mitigating one important source of uncertainty. Nevertheless, the magnitude and direction of any systematic bias remain difficult to determine, as residual uncertainty may arise from sources such as seasonal variability, weather conditions, routing, driving behaviour, data gaps, and prediction error [51]. Empirical evidence indicate that Google Maps travel time predictions are generally close to realised driving times but may, on average, be a few minutes shorter in congested settings. Optimistic bias in these estimates would lead to overstatement of accessibility (e.g., higher proportions of facilities appearing within 30- or 60-minute thresholds than in reality). However, we emphasize that existing evidence is based on very small, geographically limited studies (often with samples below 1000 trips), providing little global coverage; robust, largescale evaluations remain scarce, and future research should consider quantifying uncertainty in Google Maps travel time estimates [18, 52]. Also, finer resolution than level 14 S2 cell (approximately 600 m^2^) could improve precision but would greatly increase data-processing demands. Finally, we have not factored the actual care-seeking patterns of women using EmOC services (e.g., vehicle available, family or spousal consent and financial support) [53, 54], which is also crucial for fully characterising referral patterns. Future research should consider using actual travel time, such as was captured using a local electronic application for prehospital transport by the ambulance service in Kigali that receives emergency call outs across the city, classifies and triages responses, dispatches ambulances, and coordinates patient destinations [55]. Data from such a source will substantially improve accuracy of travel time estimation used for this kind of analysis.

For implications for policy, first, the evidence generated in this study helps to identify non-CEmOC facilities, areas and cities requiring urgent action. Our study highlighted that some facilities currently classed as non-CEmOC, if rendered functional, could bridge equity gaps in city-wide geographical access to care. In some cases, lateral referrals between CEmOC facility unnecessarily lengthens the time to definitive care [17]. Upgrading selected facilities could significantly improving maternal and newborn health outcomes. In Port Harcourt, for instance, waterlogged roads, rivers, and swampy areas exacerbate infrastructure challenges, underscoring the importance of maximum existing resources. Targeted investments to improve overall transport and road infrastructure, particularly in high-delay zones, and for particularly vulnerable populations in slums [56] could substantially reduce referral delays and enhance the efficiency of CEmOC networks. Second, our results can support the design of a coordinated system of communication and ambulance dispatch among clusters of non-CEmOC facilities sharing the same nearest public CEmOC facilities targeted at evening out referral, decreasing inter-facility travel, minimising overcrowding and ultimately improving overall access to CEmOC services. Our results can also support deployment of community-based first responders, as was done in Benin City, Nigeria [57, 58]. Third, the nearest public CEmOC (receiving) facilities of some non-CEmOC (referring) facilities are not in their own LGA, city, or even state. This, and adding those women who come to the cities for care from the rural outskirts calls for cooperation and coordination among local decision-makers and planners working across jurisdictions to design referral pathways that ensure minimal barriers when pregnant women, including when they arrive from another administrative region. In addition, our results show that peak and non-peak traffic conditions did not pose substantial difference in inter-facility travel time. This is different from our finding when we assessed journeys from home directly to CEmOC facilities [11], suggesting that any challenges experienced in connecting from a non-CEmOC facility to a public CEmOC was not principally driven by traffic. Finally, alongside identified gaps in standardised protocols and guidelines to help identify and prompt referrals [59], our findings highlight the need to standardize referral destinations and target inter-facility travel time. The 30-minute threshold for inter-facility travel time is particularly crucial as there is emerging evidence and growing consensus that it is critical benchmark for clinically significant variation in outcomes for pregnant women [13, 60, 61]. Previously, the only proposed travel time target was 2–3 h direct travel, based on postpartum haemorrhage survival time threshold [4]. However, inter-facility transfers occur after pregnant women in an emergency have engaged with the health system [62], making this the critical starting point when the public health system’s accountability for poor maternal and newborn outcomes begins. Unfortunately, many pregnant women are left on their own to travel to capable CEmOC facilities, which sometimes means doubling of travel time compared to if they had travelled directly to care [8, 9]. This is even before factoring the time it will take pregnant women to secure transport options to facilitate their referral.

## Conclusions

Inter-facility travel time to public CEmOC facilities in Nigeria varied across the highly populated cities in our study with between 6% (in Maiduguri) to 51% (in Port Harcourt) of non-CEmOC facilities being located over 30 min to their nearest public CEmOC facilities. Ensuring the shortest and most efficient transit from health facilities operating below CEmOC level plays a pivotal role in enabling pregnant women to access appropriate care when obstetric emergencies necessitate a referral. Further, the shortest travel to access appropriate care may require pregnant women to travel to contiguous cities or states. Further research is required to understand variation in service demand and utilization, and to explore the potential of cooperative efforts across levels of public health decision-making to serve a higher proportion of women and childbirths requiring obstetric referral services. Our innovative approach and findings established will inform service planning and policymaking aimed at optimising EmOC referral services in similar urban resource-constrained settings.

## Supplementary Information

Below is the link to the electronic supplementary material.

Supplementary Material 1: Approach to estimating travel time for health service accessibility studies using Google Maps

Supplementary Material 2: City maps with referral networks

Supplementary Material 3: Sensitivity analysis of random sample of 10% of non-CEmOC facilities comparing travel time estimates from Google Maps with Open Source Routing Machine and a cost-friction surface approach

## Data Availability

Datasets used for this analysis are available at: https://doi.org/10.6084/m9.figshare.22689667 [29] and https://doi.org/10.6084/m9.figshare.22699759.v1 [32]. All the codes/scripts used in the analyses presented this study are accessible via this link: https://doi.org/10.6084/m9.figshare.30394123 [36].
