# Supplementary material for: Inter-facility travel time and referral for emergency obstetric care in the 15 most populated cities of Nigeria: a spatial analysis
Source: BMC Glob Public Health. 2026 May 28;4:53. doi: 10.1186/s44263-026-00285-8 (PMC13217968; doi:10.1186/s44263-026-00285-8)
Supplement: Supplementary file 1 — Supplementary Material 1: Approach to estimating travel time for health service accessibility studies using Google Maps [file 44263_2026_285_MOESM1_ESM.pdf]

Supplementary Material 1

Approach to estimating travel time for health service accessibility studies using Google Maps

Routing/direction tools such as Google Maps Directions API (Google API), which was used in this study, aggregate data from various sources pulled within a machine learning environment to predict travel time. Main sources of data are crowdsourced data on current traffic conditions based on Global Positioning System activated on mobile phones, agile data based on historic traffic patterns anonymously captured and stored over time, with emphasis on past two to four weeks (a recent change to estimation approach after the COVID-19 pandemic), authoritative data from local governments on speed limits, tolls, or a restriction is in force, and real-time feedback from drivers regarding weather conditions, accidents, or constructions. By relaying anonymous mobile phone's location, the time it takes to move from one location to another can be computed. The estimated time of arrival predictions from the Google API have a very high accuracy bar, with the predictions consistently accurate for over 97% of trips as it depends on live traffic data to govern travel speeds (indirectly accounting for congestion due to different factors, time of the day, and day of the week when the journey was made) along a route.

Figure S1. Process of travel time extraction for health service accessibility studies using Google Maps

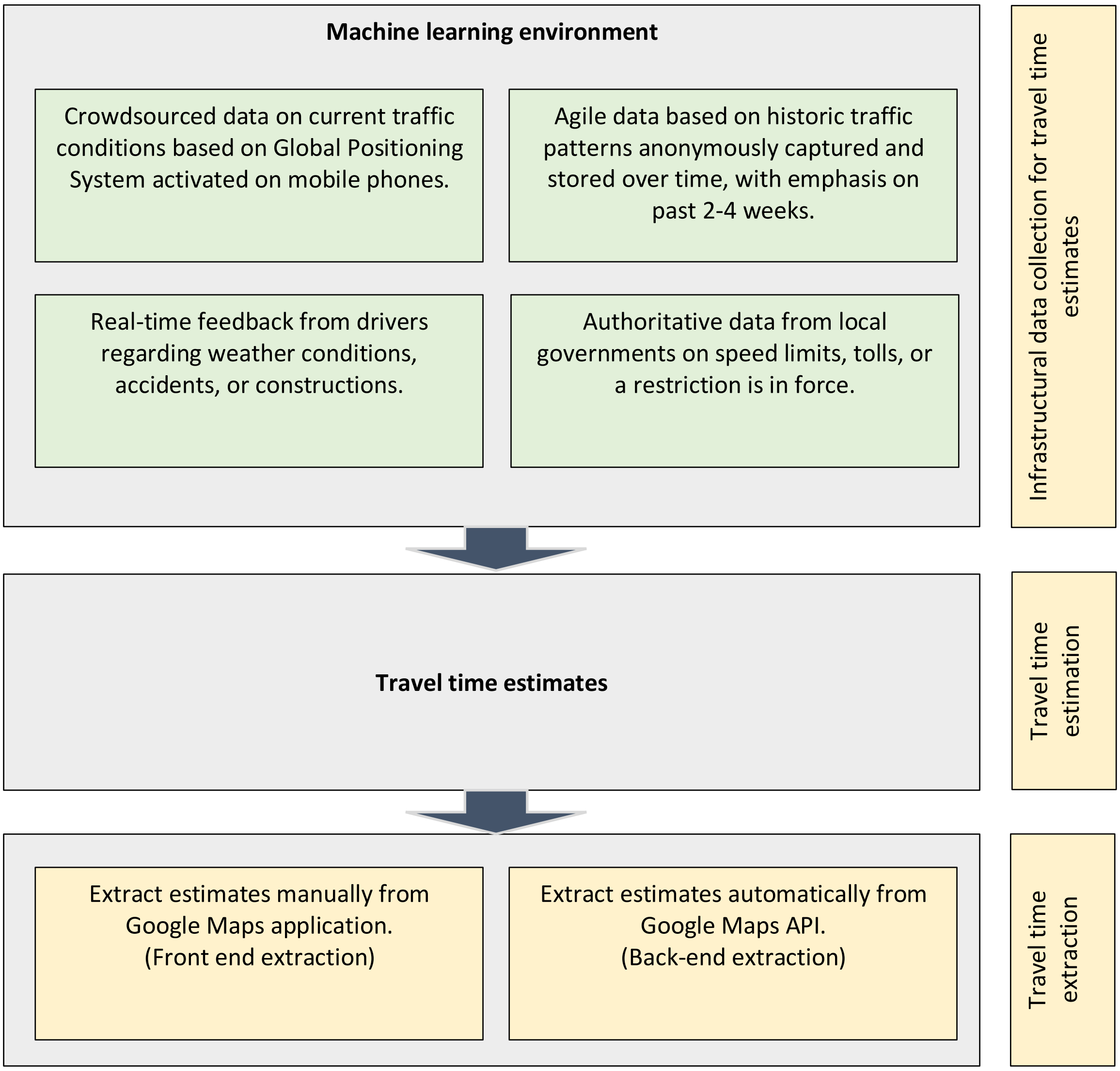

When done from the front end, an actual journey needs to be entered into Google Maps via the Google Maps website or using the mobile app version. This approach has been used in studies conducted in Dhaka, Bangladesh,[1] Lagos, Nigeria,[2] and Nairobi, Kenya. However, from the back end, people requesting directions via this API do not need to undertake a journey for this API to work - the API estimates travel times based on variables, as described above. Google Maps' Directions API underpins Google Maps as it is the same API that also allows users to plan

journeys when start and end points are inputted on the Google Maps website or in the mobile app. The API provides directions given a start point and an end point, but no actual travel is needed to get results from the API.

There are Internal and External versions of the API, both of which have been used on different projects. In this study, we used the Internal version. However, the external version has also been used in research (for example, in Cali, Colombia).[3] Both versions are identical and will produce the same travel times as each other and from what you would get from inputting a starting point and destination in the actual Google Maps App. The only difference is in the usage. The use of that API is governed by a Terms of Service that does not allow cache-ing (e.g., storing data so that future requests for that data can be served faster). However, the Internal version of the API can be cached. The usage of the API allows us to leverage Google travel time estimates to take into consideration historical travel and traffic patterns, time of day and additional considerations baked into their model. When traffic is particularly heavy, Google may advise users navigating through the area of alternate routes they can take to avoid traffic and save time. However, this method requires an accumulation of traffic, which takes time, and can therefore mean drivers still get caught up in queues.

This is just one example, of which there are almost limitless possibilities.

More information on how Google estimates travel time can be accessed via the resources listed below:

Lau J (2020) Google Maps 101: How AI helps predict traffic and determine routes. Available at: https://blog.google/products/maps/google-maps-101-how-ai-helps-predict-traffic-and-determine-routes/

Shashidharan S (2023) How AI and imagery keep speed limits on Google Maps updated. Available at: https://blog.google/products/maps/how-ai-and-imagery-keep-speed-limits-on-google-maps-updated/

Barth D (2009) The bright side of sitting in traffic: Crowdsourcing road congestion data. Available at: https://electronics.howstuffworks.com/how-does-google-maps-predict-traffic.htm

Clough (2023) AI Maps - How is AI being used in Mapping in 2023?
https://www.pointr.tech/blog/ai-maps-how-is-ai-being-used-in-mapping#:~:text=With%20AI%2C%20systems%20such%20as,before%20they've%20even%20happened.

References

1. Zafri NM, Afroj S, Ali MA, Hasan MMU, Rahman MH. Effectiveness of containment strategies and local cognition to control vehicular traffic volume in Dhaka, Bangladesh during COVID-19 pandemic: Use of Google Map based real-time traffic data. PLoS One. 2021;16(5):e0252228.
2. Banke-Thomas A, Wong KLM, Collins L, Olaniran A, Balogun M, Wright O, et al. An assessment of geographical access and factors influencing travel time to emergency obstetric care in the urban state of Lagos, Nigeria. Health Policy Plan. 2021;36(9):1384–96.
3. Cuervo LG, Martinez-Herrera E, Osorio L, Hatcher-Roberts J, Cuervo D, Bula MO, et al. Dynamic accessibility by car to tertiary care emergency services in Cali, Colombia, in 2020: cross-sectional equity analyses using travel time big data from a Google API. BMJ Open. 2022;12(9):e062178.
4. Brinkhoff T. Federal Republic of Nigeria [Internet]. City population. 2022 [cited 2024 Jan 24]. Available from: https://www.citypopulation.de/en/nigeria/
5. Dania F. Lagos's Resilience Journey [Internet]. Resilient cities network. 2022 [cited 2024 Jan 13]. Available from: https://resilientcitiesnetwork.org/lagos/
