# Supplementary material for: Inter-facility travel time and referral for emergency obstetric care in the 15 most populated cities of Nigeria: a spatial analysis
Source: BMC Glob Public Health. 2026 May 28;4:53. doi: 10.1186/s44263-026-00285-8 (PMC13217968; doi:10.1186/s44263-026-00285-8)
Supplement: Supplementary file 2 — Supplementary Material 2: City maps with referral networks [file 44263_2026_285_MOESM2_ESM.pdf]

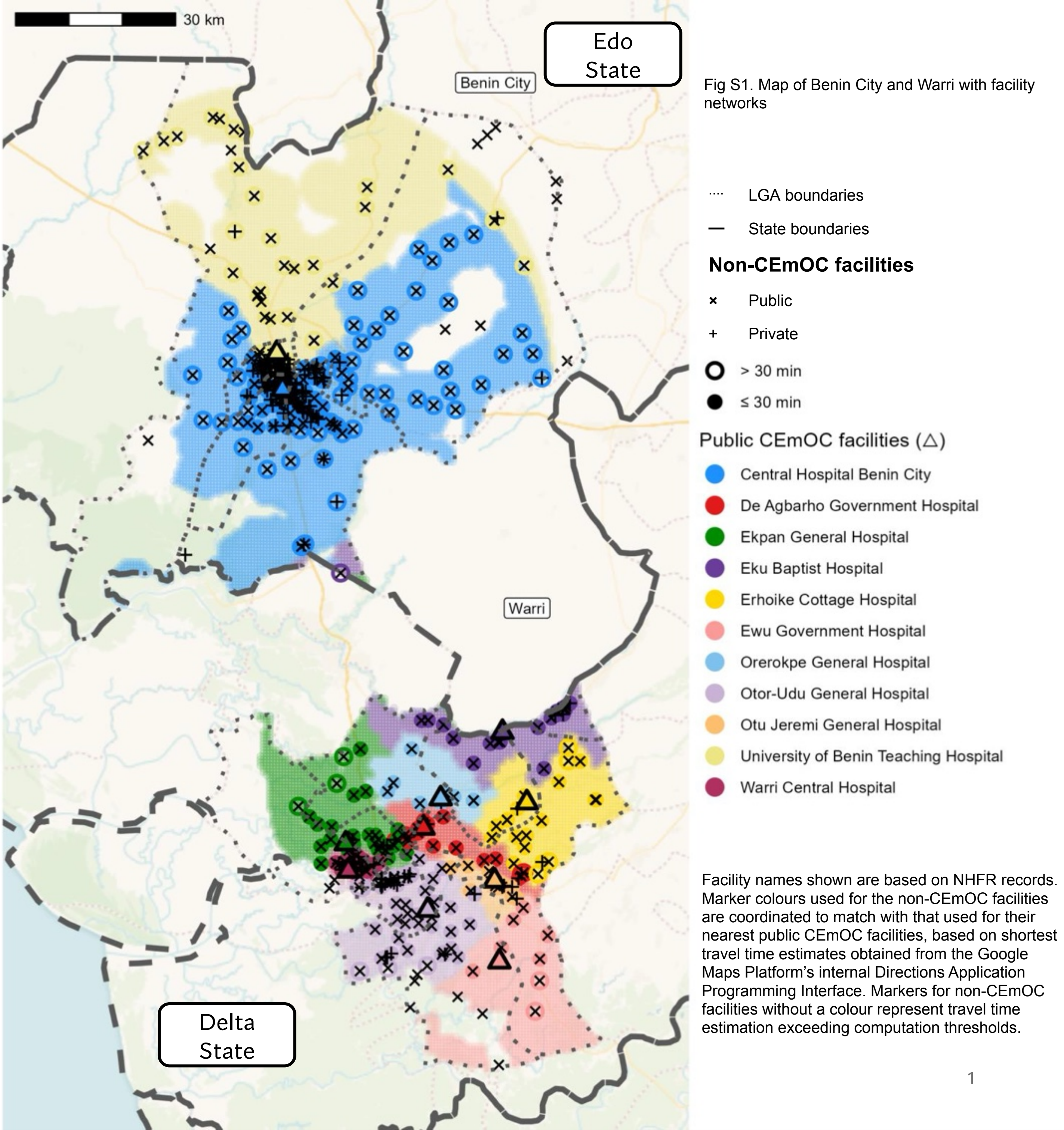

Fig S1. Map of Benin City and Warri with facility networks

Facility names shown are based on NHFR records. Marker colours used for the non-CEmOC facilities are coordinated to match with that used for their nearest public CEmOC facilities, based on shortest travel time estimates obtained from the Google Maps Platform's internal Directions Application Programming Interface. Markers for non-CEmOC facilities without a colour represent travel time estimation exceeding computation thresholds.

Fig S2. Map of Aba, Onitsha, Owerri, Port Harcourt, and Uyo with facility networks

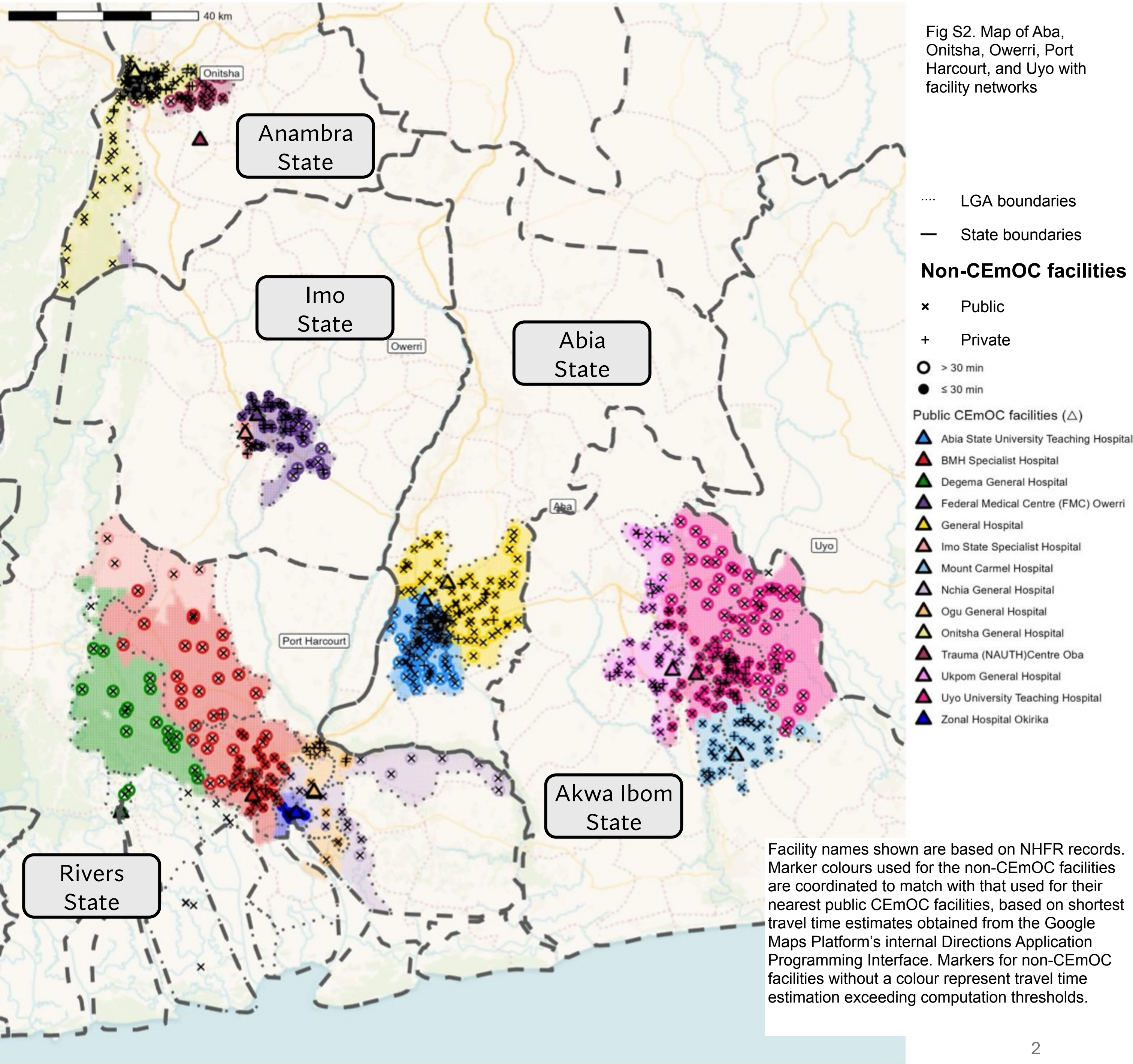

Facility names shown are based on NHFR records. Marker colours used for the non-CEmOC facilities are coordinated to match with that used for their nearest public CEmOC facilities, based on shortest travel time estimates obtained from the Google Maps Platform's internal Directions Application Programming Interface. Markers for non-CEmOC facilities without a colour represent travel time estimation exceeding computation thresholds.

Facility names shown are based on NHFR records. Marker colours used for the non-CEmOC facilities are coordinated to match with that used for their nearest public CEmOC facilities, based on shortest travel time estimates obtained from the Google Maps Platform's internal Directions Application Programming Interface. Markers for non-CEmOC facilities without a colour represent travel time estimation exceeding computation thresholds.

Fig S3. Map of Lagos with facility networks

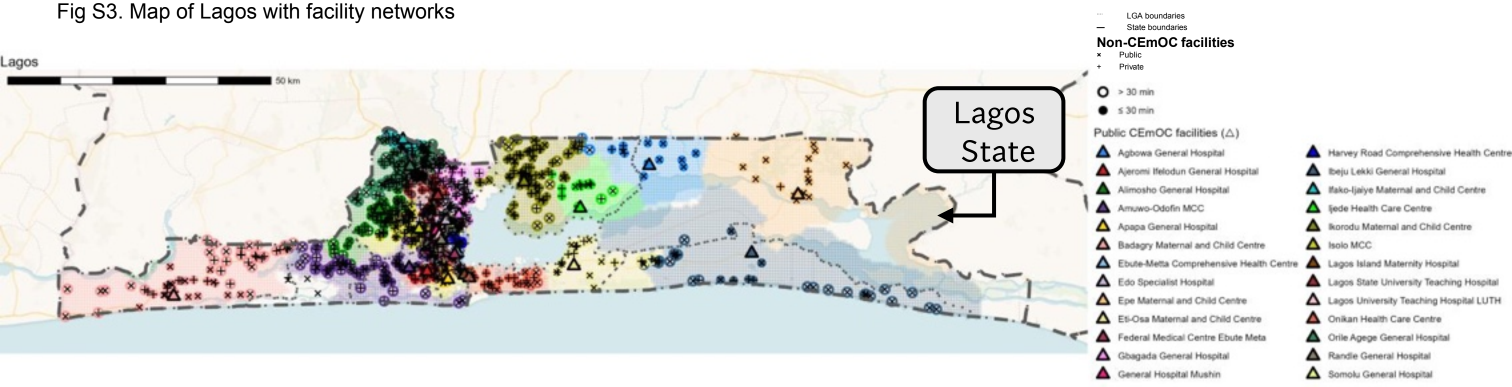

Fig S4. Map of Abuja with facility networks

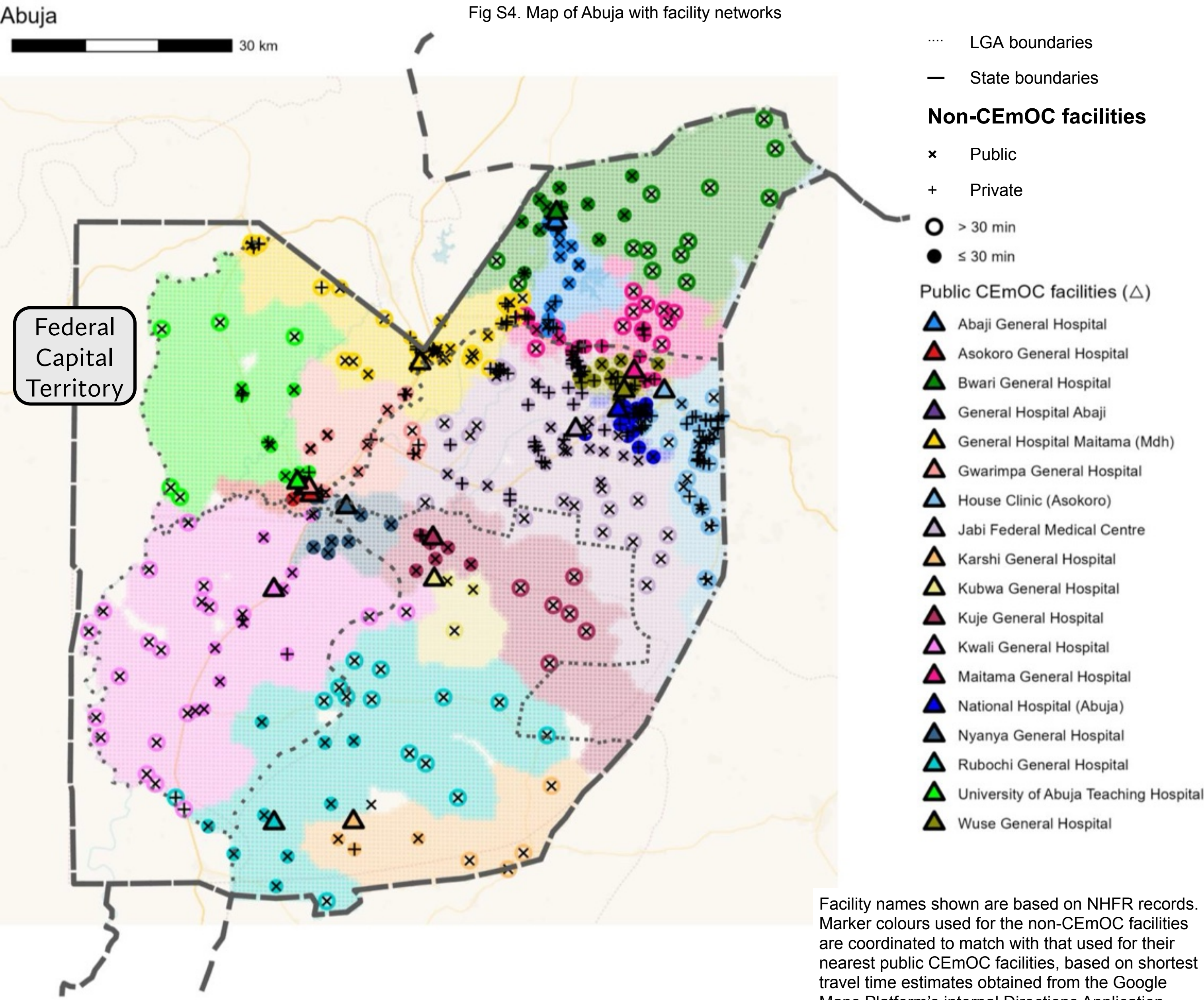

Facility names shown are based on NHFR records. Marker colours used for the non-CEmOC facilities are coordinated to match with that used for their nearest public CEmOC facilities, based on shortest travel time estimates obtained from the Google Maps Platform's internal Directions Application Programming Interface. Markers for non-CEmOC facilities without a colour represent travel time estimation exceeding computation thresholds.

Fig S5. Map of Ibadan with facility networks

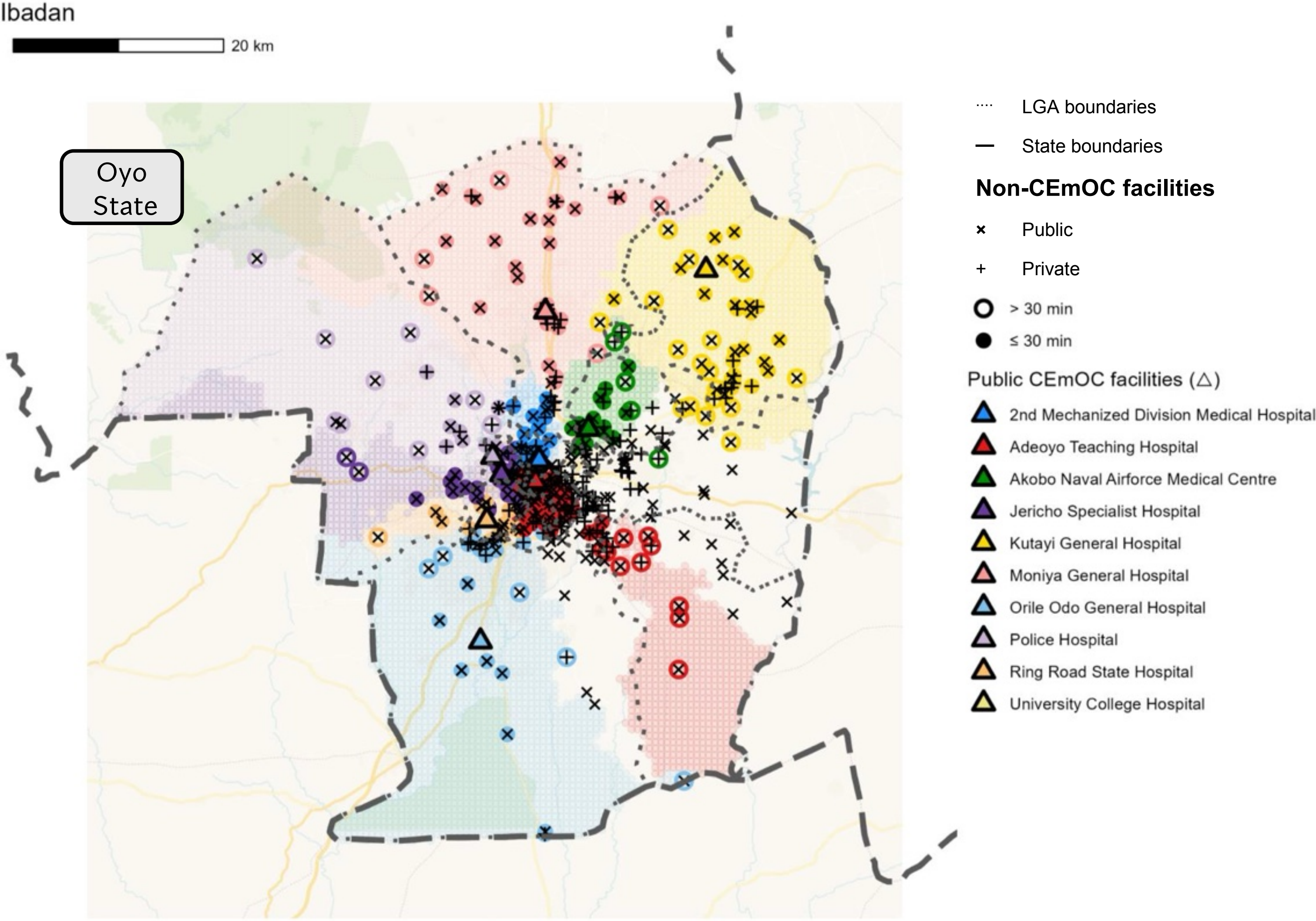

Facility names shown are based on NHFR records. Marker colours used for the non-CEmOC facilities are coordinated to match with that used for their nearest public CEmOC facilities, based on shortest travel time estimates obtained from the Google Maps Platform's internal Directions Application Programming Interface. Markers for non-CEmOC facilities without a colour represent travel time estimation exceeding computation thresholds.

Fig S6. Map of Ilorin with facility networks

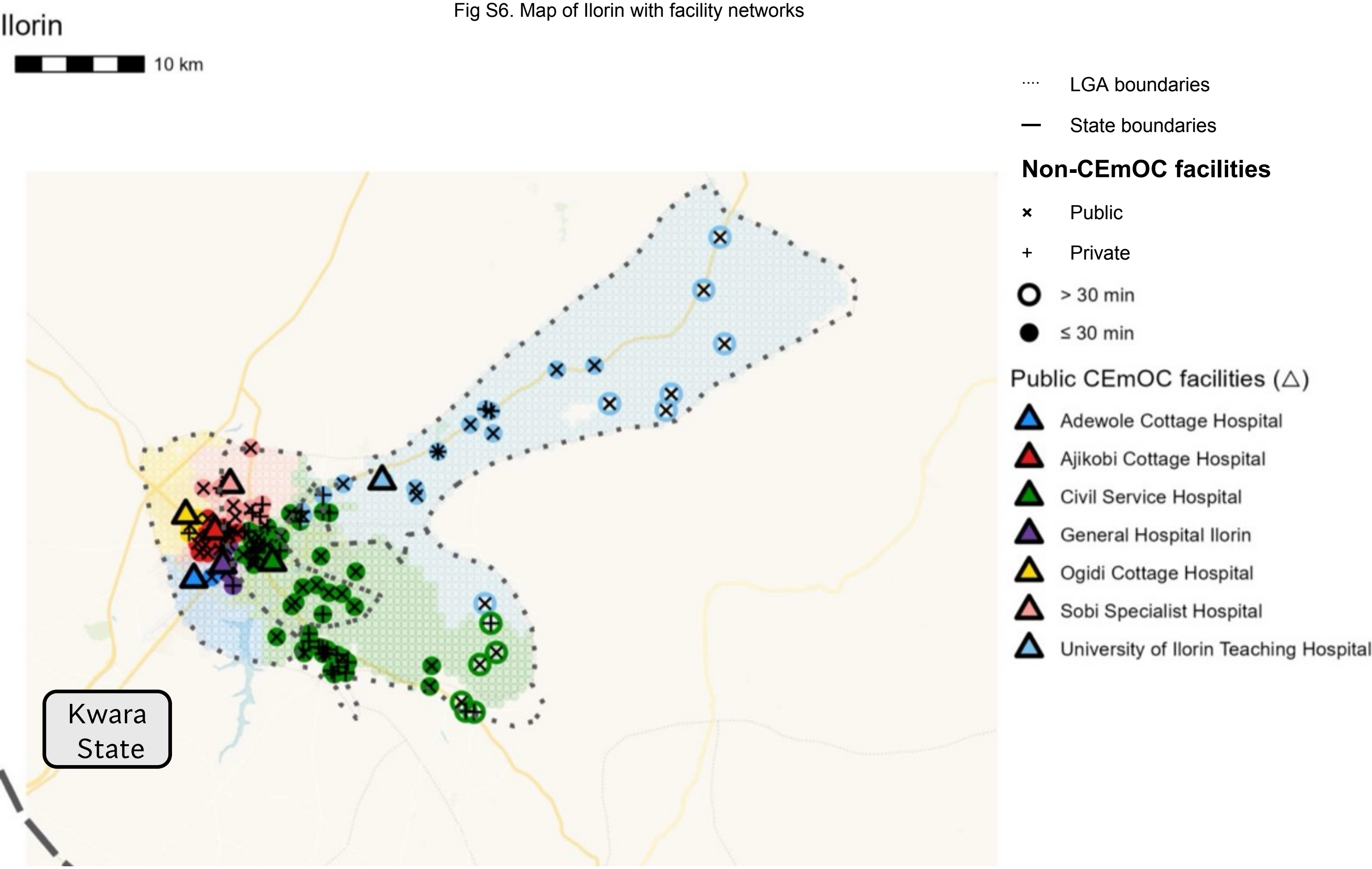

We note that the Maternity Wing of University of Ilorin Teaching Hospital is located in a different local government area from the main hospital.

Facility names shown are based on NHFR records. Marker colours used for the non-CEmOC facilities are coordinated to match with that used for their nearest public CEmOC facilities, based on shortest travel time estimates obtained from the Google Maps Platform's internal Directions Application Programming Interface. Markers for non-CEmOC facilities without a colour represent travel time estimation exceeding computation thresholds.

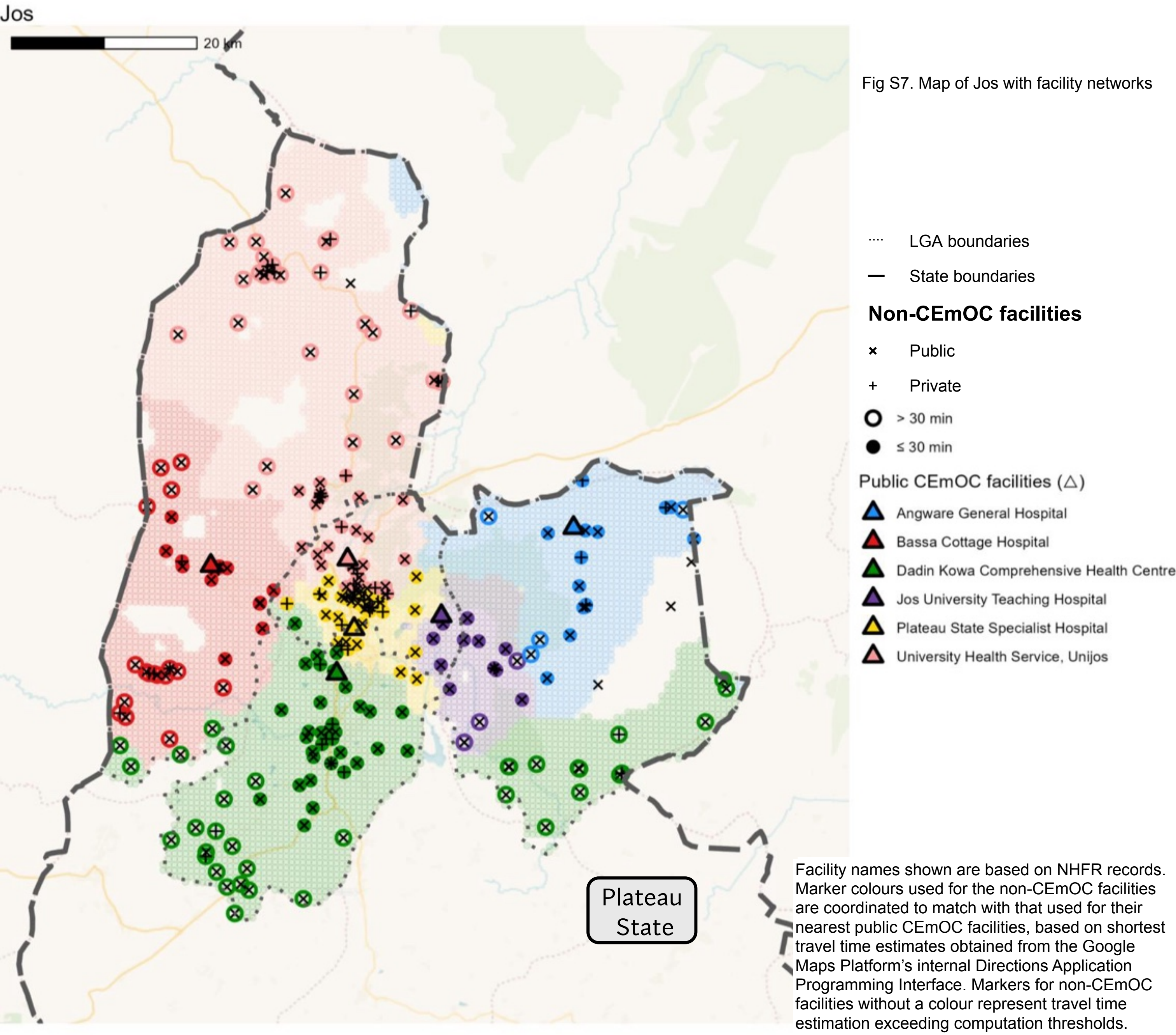

Fig S7. Map of Jos with facility networks

Facility names shown are based on NHFR records. Marker colours used for the non-CEmOC facilities are coordinated to match with that used for their nearest public CEmOC facilities, based on shortest travel time estimates obtained from the Google Maps Platform's internal Directions Application Programming Interface. Markers for non-CEmOC facilities without a colour represent travel time estimation exceeding computation thresholds.

Fig S8. Map of Kano with facility networks

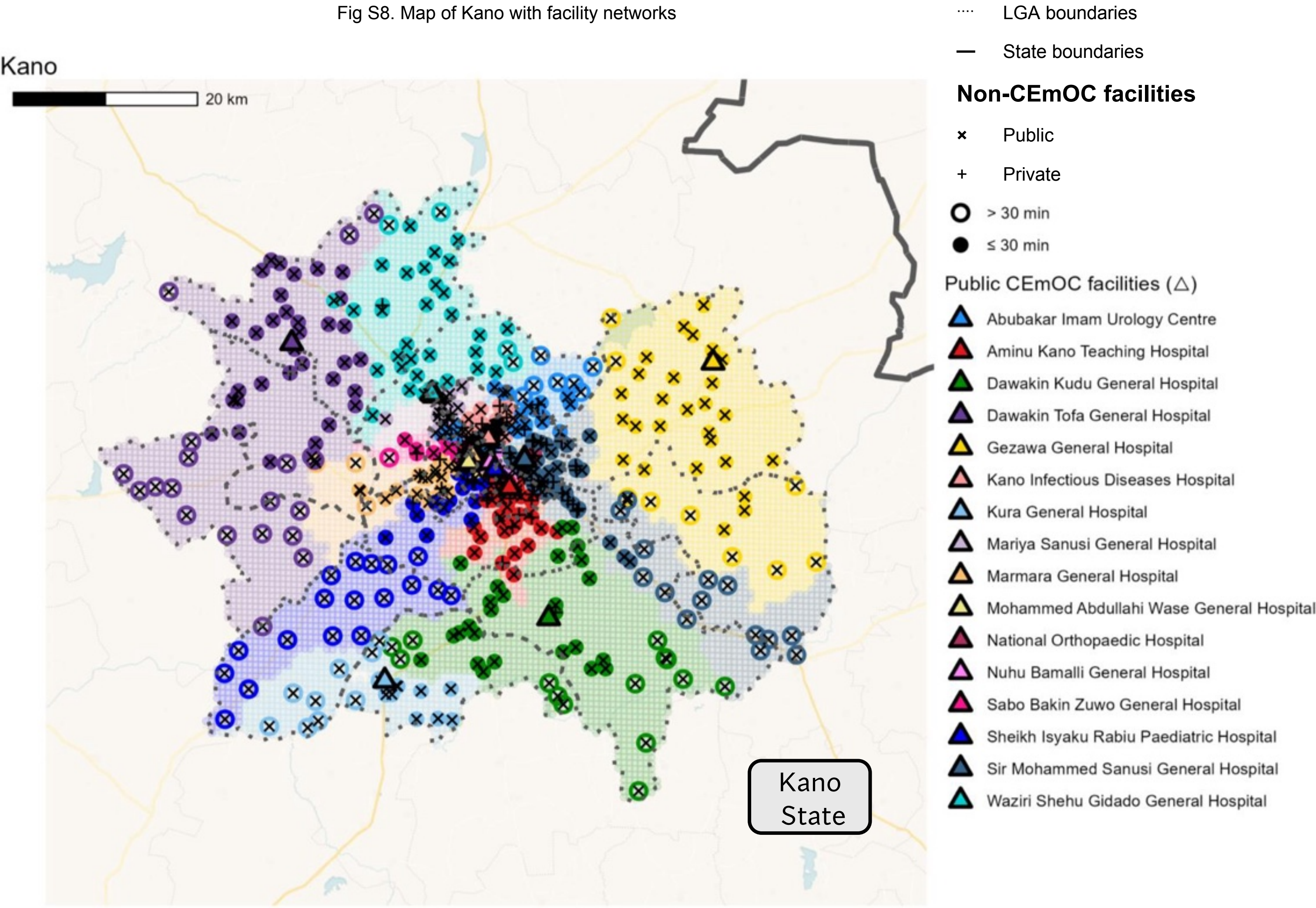

Facility names shown are based on NHFR records. Marker colours used for the non-CEmOC facilities are coordinated to match with that used for their nearest public CEmOC facilities, based on shortest travel time estimates obtained from the Google Maps Platform's internal Directions Application Programming Interface. Markers for non-CEmOC facilities without a colour represent travel time estimation exceeding computation thresholds.

Fig S9. Map of Maiduguri with facility networks

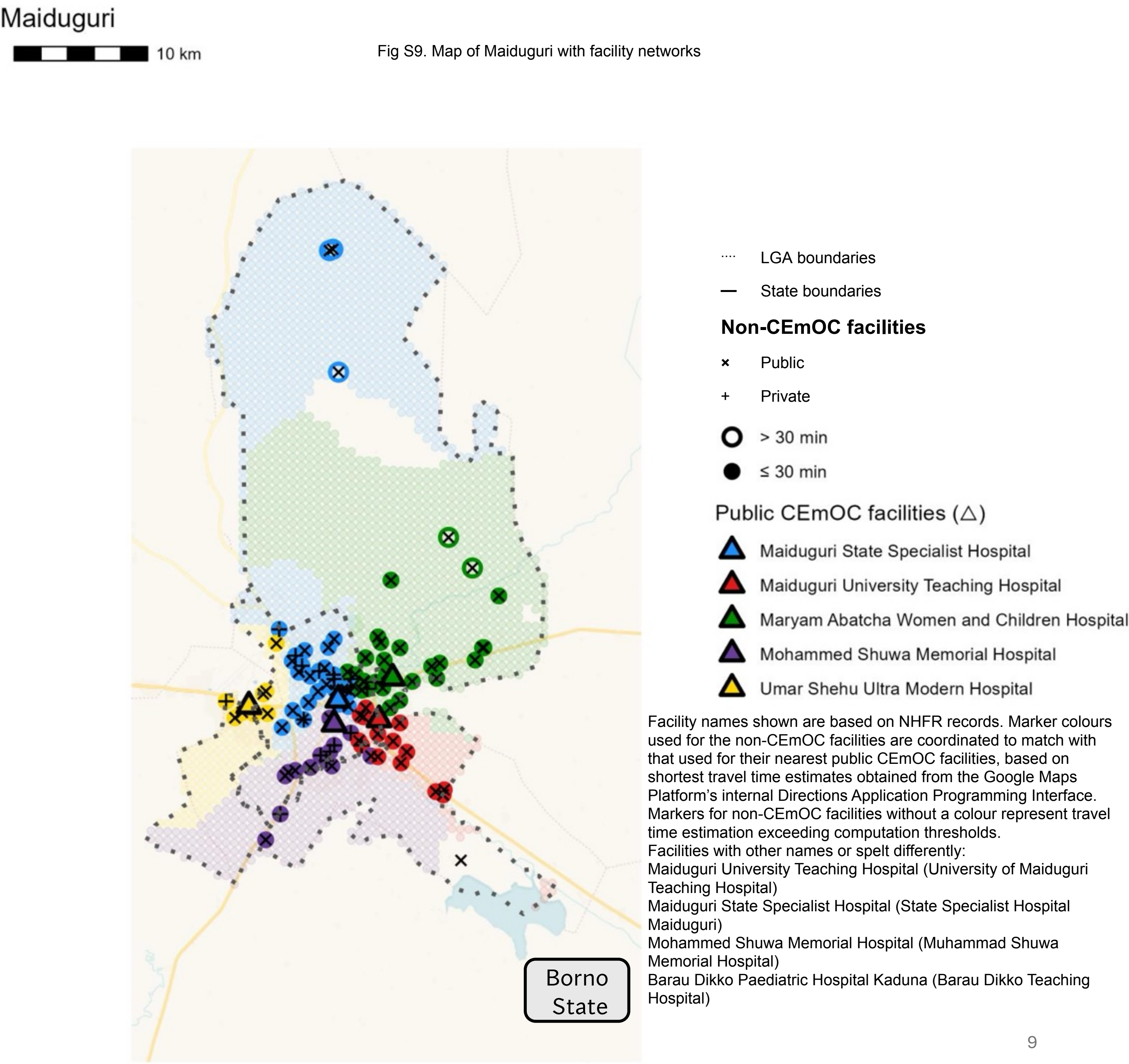

Facility names shown are based on NHFR records. Marker colours used for the non-CEmOC facilities are coordinated to match with that used for their nearest public CEmOC facilities, based on shortest travel time estimates obtained from the Google Maps Platform's internal Directions Application Programming Interface. Markers for non-CEmOC facilities without a colour represent travel time estimation exceeding computation thresholds.

Facilities with other names or spelt differently:

Maiduguri University Teaching Hospital (University of Maiduguri Teaching Hospital)

Maiduguri State Specialist Hospital (State Specialist Hospital Maiduguri)

Mohammed Shuwa Memorial Hospital (Muhammad Shuwa Memorial Hospital)

Barau Dikko Paediatric Hospital Kaduna (Barau Dikko Teaching Hospital)

Fig S10. Map of Kaduna with facility networks

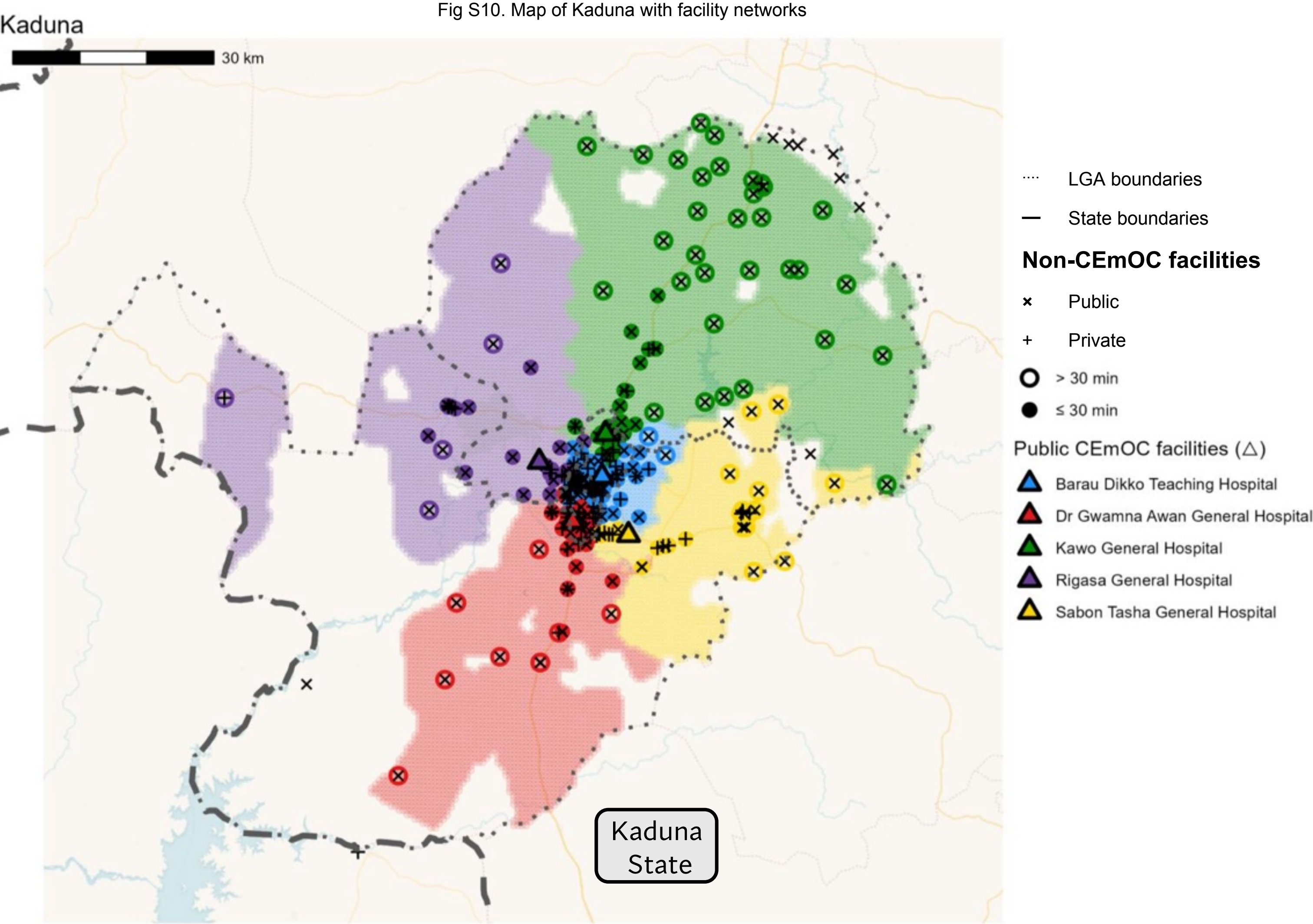

Facility names shown are based on NHFR records. Marker colours used for the non-CEmOC facilities are coordinated to match with that used for their nearest public CEmOC facilities, based on shortest travel time estimates obtained from the Google Maps Platform's internal Directions Application Programming Interface. Markers for non-CEmOC facilities without a colour represent travel time estimation exceeding computation thresholds.
Facilities with other names or spelt differently:
Barau Dikko Paediatric Hospital Kaduna (Barau Dikko Teaching Hospital)
Bassa Cottage Hospital (Wase Cottage Hospital)
